# Antioxidant properties in some selected cyanobacteria isolated from fresh water bodies of Sri Lanka

**DOI:** 10.1002/fsn3.340

**Published:** 2016-01-20

**Authors:** Md Fuad Hossain, R. R. Ratnayake, Kirisnashamy Meerajini, K. L. Wasantha Kumara

**Affiliations:** ^1^ National Institute of Fundamental StudiesHantana RoadKandySri Lanka; ^2^ Department of Agricultural BiologyFaculty of AgricultureUniversity of RuhunaMapalanaKamburupitiyaMataraSri Lanka; ^3^ Faculty of Science and TechnologyUvaWellassa UniversityBadullaSri Lanka

**Keywords:** Antioxidant, cyanobacteria, DPPH, FRAP, phycobiliproteins, total flavonoid content, total phenolic content

## Abstract

Phytonutrients and pigments present in cyanobacteria act as antioxidants, which facilitate the formation of body's defense mechanism against free radical damage to cells. The aim of this investigation was to study the total phenolic content (TPC), total flavonoid content (TFC), antioxidant activity, phycobiliproteins (PBPs), and active compounds in four cyanobacterial species, that is, *Oscillatoria* sp., *Lyngbya* sp., *Microcystis* sp., and *Spirulina* sp. isolated from fresh water bodies of Sri Lanka. In this study, *Lyngbya* sp.*,* showed highest TPC (5.02 ± 0.20 mg/g), TFC (664.07 ± 19.76 mg/g), and total PBPs (127.01 mg/g) value. The ferric reducing antioxidant power (FRAP) was recorded highest in *Oscillatoria* sp. (39.63 ± 7.02), whereas the 2,2‐diphenyl‐1‐picrylhydrazyl (DPPH) radical scavenging activity was also reported the highest in *Oscillatoria* sp. (465.31 ± 25.76) followed by *Lyngbya* sp. (248.39 ± 11.97). In FTIR spectroscopy, *Lyngbya* sp. does not show any N‐H stretching band which is ultimately responsible for the inhibition of antioxidant activity. The study revealed that *Lyngbya* sp. and *Oscillatoria* sp. can be an excellent source for food, pharmaceutical, and other industrial uses.

## Introduction

Cyanobacteria have a significant attraction as natural source of bioactive molecules with a broad range of biological activities including antimicrobial, antiviral, anticancer, antioxidant and anti‐inflammatory effects (Tuney et al. 2006; Patra et al. 2009). Cyanobacteria believed to be rich in antioxidants and phycobiliproteins (Mata et al. 2010) (PBPs) which are the unique photosynthetic pigments of cyanobacteria. These pigments have been widely used as natural colorants in foods, cosmetics, and pharmaceuticals particularly as substitutes for synthetic dyes (Singh et al. 2005). In addition, PBPs are also used in the field of immunology due to their fluorescent properties. The unique character of high molar absorbance coefficients, high fluorescence quantum yield, large stokes shift, high oligomer stability, and also their high photostability make phycobiliproteins highly efficient fluorescent substances (Spolaore et al. 2006). Free radicals released during oxidative stress pose the major endogenous damage in the biological system (Thajuddin and Subramanian 2010). This type of damage is often associated with various degenerative diseases and disorders such as cancer, cardiovascular disease, immune function decline, and aging. Besides damage to living cells, free radicals are the major cause of food deterioration through lipid oxidation, which ultimately affects the organoleptic properties and edibility of foods (Huang et al. 2007). To counter the effects of oxidative stress, many people consume antioxidants in the form of commercial food additives that have been manufactured synthetically and may contain high amounts of preservatives(Shasha et al. 2014). But, most of the sources of antioxidants reported till date are competitive with traditional foods and crops. However, their rapid production and usage for industrial and medical purposes are not easy and their attainment of optimal growth takes a longer time(Nakao et al. 2009; Aydas¸ et al. 2013). Therefore, to find out a nonconventional and unexplored source of antioxidant such as cyanobacteria will create a vast opportunity in this field. Because cyanobacteria is noncompetitive with traditional food crops, can double in volume overnight and can be harvested on daily basis. In contrast, cyanobacteria blooms are frequently associated with toxin production. In the last decades, the amount of papers published on the diverse cyanotoxins has been increasing with microcystins (MC) as the winners with cylindrospermopsin (CYN) as the second runner (Vasconcelos 2015). So, selection of correct species for the source of antioxidant for industrial use is of great concern. The aim of this investigation was to study the antioxidant properties, and to find out a correlation with active compounds and antioxidant properties in selected cyanobacteria isolated from fresh water bodies of Sri Lanka.

## Materials and Methods

### Sample collection and preparation

Water samples were collected from wet zone & dry zone of Sri Lanka using a Ruttner sampler from the photic surface layer of the reservoir and then kept in a plastic can till the sample was prepared for culturing. The photic zone of the water was determined by the Secchi disk dipping into the water from the surface area. From each sample, 2 L was filtered through 20 *μ*m mesh size planktonic net. The retentiate was transferred into a screw cap plastic tube making the final volume into 25 mL by adding original water. A quantity of 10 mL of the planktonic samples were transferred into 50 mL of BG11 (Stanier et al. 1971) and GO (BG11‐N_0_) (Rippka et al. 1979) media in 100 mL conical flask for culturing.

### Culturing and subculturing

Samples with media were kept on the shaker in biological growth chamber under 2000 lux light intensity at pH 7.5 and 200 rpm of shaking. Growth appearance was expressed as days in each media for the cultures to appear the bluish green color. Frequent subculturing was practiced to isolate single colonies from mixed growths of microorganisms (Pulz and Gross 2004). In case of filamentous cyanobacteria when it was very difficult to separate them, a vortex mixture was used. A quantity of 1 mL of the sample, some pieces of glass beads were poured into a microcentrifuge tube and was vortexed. Subcultures were carried out in petri plate containing media solidified with 1.5% (w/v) agar.

### Morphological identification

Monoculture of the sample was put on a glass slide, covered with a cover slip and observed under the microscope. Identifications were carried out using morphological characteristics described by Desikachary (1959).

### Biomass harvest

Algal biomass was harvested by centrifuging the cultures in 1075 g for 5 min at 27°C temperature. The cell pellet was separated in order to be dried at 55°C for 24 h in oven to obtain dry matter (%).

### Preparation of extracts

A quantity of 0.15 g of dry biomass was taken with 10 mL of distilled water and cell disruptions were carried out using the process of sonication (Sonics, vibra‐cell TM, USA) at 10 KHz for 5 min (Sharathchandra and Rajashekhar 2013). After cell disruption, any insoluble material was removed from the cell‐free extract by centrifugation with 2000 rpm at 27°C for 5 min. The supernatant were obtained for analysis.

### Phytochemical analysis

Total phenolic content (TPC) was determined using microplate reader by the method of Chandler and Dodds, 1983 (Chandler and Dodds 1983) with some modification. Briefly, 40 *μ*L of the different cell‐free extracts of the four cyanobacteria was mixed with 25 *μ*L of distilled water, 105 *μ*L of 10% Folin–Ciocalteu reagent (100 *μ*L Folin mixed with 900 *μ*L of distilled water). After a 3 min incubation, 80 *μ*L of 7.5% (w/v) sodium carbonate was added and the mixture was then incubated for 30 min. The absorbance at 760 nm was measured. The concentrations of TPC were determined as gallic acid equivalents (GAEs mg/g of dry weight).

Total flavonoid content (TFC) was determined using microplate method described by Thaipong et al.*,* 2006 (Thaipong et al. 2006) with some modification. In brief, a 0.5 mL of each extract was taken. Afterwards, 0.15 mL of 5% NaNO_2_ solution followed by 2 mL distilled water were mixed and was left for 6 min. Thereafter, 0.15 mL of (10%) AlCl_3_ solution was added and allowed to stand for 6 min. A quantity of 2 mL of (4%) NaOH solution was added to the mixture and the final volume was adjusted to 5 mL with distilled water. The mixture was thoroughly shaken and allowed to stand for 15 min. Absorbance of the reaction mixture was read at 510 nm. The concentrations of total flavonoids were determined as quercetin equivalents (QEs mg/g of dry weight). All determinations were carried out in triplicate.

### Antioxidant activity

The scavenging activity of the different preheated cell‐free extract samples of the four cyanobacteria on 2,2‐diphenyl‐1‐picrylhydrazyl (DPPH) radical scavenging activity was measured according to the method of Hou et al., 2001(Hou et al. 2001) with some modification. DPPH concentration was prepared as 0.06 mg/mL in methanol. A quantity of 60 *μ*L of the different cell‐free extract samples and 90 *μ*L distilled water followed by 100 *μ*L DPPH solution was added. Then, it was kept for 30 min under light protection. The absorbance at 517 nm was taken. An ascorbic acid (AA) standard curve was plotted (Calculation formula as Y = −0.1704X + 0.7516 where, *R* ^*2* ^= 0.9865) using the standard solution (0.01 mg m/L). All determinations were carried out in triplicate.

The ferric reducing antioxidant power (FRAP) assay was performed according to the method of Benzie and Strain, 1999(Benzie and Strain 1999) with some modification. At low pH, the reduction of ferric tripyridyltriazine complex to the ferrous form produces an intense blue color that can be monitored by measuring the absorbance at 593 nm. Briefly, 200 *μ*L of the FRAP solution was mixed with 50 *μ*L of sample. The reaction mixture was incubated at 37°C for 4 min and the absorbance was measured at 593 nm. The FRAP reagent was prewarmed at 37°C and was always freshly prepared by mixing 10 volumes of 300 mmol/L acetate buffer (pH 3.6) with one volume of 10 mmol/L TPTZ solution dissolved in 10 mmol/L HCl and one volume of 20 mmol/L FeCl_3_.6H_2_O. A calibration curve was prepared using an aqueous solution of ferrous sulfate FeSO4.7H_2_O (7.5 to 720 *μ*mol/L). FRAP values were expressed as micromoles of ferrous equivalent (*μ*mol/L Fe[II]) per g of sample.

### Quantitative analysis of phycobiliproteins

The phycobiliprotein contain phycoerythrin (PE), phycocyanin (PC), and allophycocyanin (APC). The phycobiliprotein content was measured using 1 mL of different samples of cell‐free extracts of the four cyanobacteria by spectrophotometer. PE was measured at 562 nm, PC at 615 nm, and APC at 652 nm. The quantities of PE, PC, and APC in the different extracts, and biliprotein containing solutions were calculated from the measurement of absorbance at 562, 615, and 652 nm using the following equations (Bennett and Bogorad 1973; Bryant et al. 1979):

$$\text{APC}\;=\;\left\{A_{652}-0.208\left(A_{615}\right)\right\}/5.09\;\text{mg}\;{\text{mL}}^{-1}$$

$$\text{PC}\;=\;\left\{A_{615}-0.474\left(A_{652}\right)\right\}/5.34\;\text{mg}\;{\text{mL}}^{-1}$$

$$\text{PE}\;=\;\left\{A_{562}-2.41\left(\text{PC}\right)-0.849\left(\text{APC}\right)\right\}/9.62\;\text{mg}\;{\text{mL}}^{-1}$$

### Active compound analysis

Using the FTIR spectroscopy, the active compounds were identified(Mistry 2009). A quantity of 1.5 *μ*L of sample was taken into a microcentrifuge tube and placed into the vacuum desiccator for 72 h to evaporate the moisture. Then, the remaining samples were used for FTIR analysis.

### Statistical analysis

Statistical analysis were done using ANOVA in SAS 9.1 (SAS, 1999), MINITAB‐14, and SPSS‐16 statistical software packages. Data were presented as mean ± standard deviation (SD) of three replicates. The *P*‐values less than 0.05 were considered significant.

## Result and Discussion

### Morphological identification

Four cyanobacteria species were isolated and identified as *Oscillatoria* sp*. Lyngbya* sp*. Microcystis* sp*.,* and *Spirulina* sp., in this study. The morphological characteristics for *Oscillatoria* sp. were recorded as filamentous with trichome organization, without distinct sheath, without branching, absence of heterocyst, and no false branching. Characteristics for *Lyngbya* sp. were filamentous with trichome organization and distinct sheath, each sheath with single trichome, lamellate, brownish color without branching, and heterocyst absent. However, *Microcystis* sp. was identified as unicellular, colonies with closely packed cells, and homogenous sheath, individual cell does not have a sheath, cells without any regular arrangement within the colony. The characteristics for *Spirulina* sp. were recorded as trichomes multicellular cylindrical, sheath absent, trichomes coiled into a regular spiral shape, apex of trichome were not attenuated, terminal cell rounded, and without calyptra. Some species of *Microcystis* is a potential toxin‐producing agent (Ruangsomboon et al. 2014), whereas most of the species from *Oscillatoria, Lyngbya, Microcystis*, and *Spirulina* are reported for antimicrobial (Padhi et al. 2014; Yadav et al. 2015), pharmaceutical (Raja et al. 2015), waste water treatment (Sood et al. 2015), and biofuel industry (Thingujam et al. 2015).

### Phytochemical analysis

Polyphenols serve as powerful antioxidants due to the hydrogen‐donating ability of their hydroxyl groups as well as their ability to donate electrons to arrest the production of free radicals as a result of oxidative stress (Afroz et al. 2014). Flavonoids are the largest class of polyphenols, with a common diphenylpropane structure (C6–C3–C6) consisting of two aromatic rings linked by three carbons. The mechanisms of action of flavonoids are exerted via scavenging or chelating processes (Schmitt‐Schillig et al. 2005). In this study, *Lyngbya* sp. contain high concentration of TPC (5.02 ± 0.20, GAEs mg/g) and TFC (664.07 ± 19.76 QEs mg/g) (Table 1). The TPC value reported in *Lyngbya* sp. is higher than some other cyanobacteria which is reported from different studies. A study carried out by Sharathchandra and Rajashekhar (2013) reported highest TPC value in *Lyngbya limnetica* (355 mg/g) followed by *Calothrix fusca (*260 mg/g), *Phormidium fragile* (180 mg/g), and *Scytonema bohneri* (145 mg/g) (Sharathchandra and Rajashekhar 2013). Another study carried out on four thermotolerant cyanobacteria showed that the TPC value in *Lyngbya* sp. which is reported in this study is higher than *Scytonema* sp. TP40 (2.75 ± 0.08 mg/g) and *Cyanosarcina* sp. SK40 (1.88 ± 0.04 mg/g) but lower than *Leptolyngbya* sp. KC45 (6.24 ± 0.06 mg/g) and *Phormidium* sp. PD40‐1 (5.43 ± 0.01 mg/g) (Pumas et al. 2011). A study carried out by Aydas et al., reported the TPC value in *Synechocystis* sp. BASO444 (66.0 ± 1.2 *μ*g/g) and *Synechocystis* sp. BASO673 (78.1 ± 1.8 *μ*g/g) (Aydas¸ et al. 2013). Also, all four cyanobacteria species selected for this study contain higher flavonoid content (Table 1) than that of all four species selected for the previous study (Sharathchandra and Rajashekhar 2013). Flavonoids have been reported to have anti‐inflammatory and antimicrobial activities and can also inhibit platelet aggregation and the release of mast cell histamine (Koley et al. 2011). In this regard, cyanobacteria may have important health benefits.

**Table 1:** Antioxidant value of different cyanobacteria strains

Species	TPC (GAEs mg/g)	TFC (QEs mg/g)	DPPH scavenging activity(AAs mg/g)	FRAP (*μ*M Fe[II]/100 g)
*Oscillatoria* sp.	2.96 ± 0.14^d^	552.59 ± 46.27^d^	465.31 ± 25.76^d^	39.63 ± 7.02^d^
*Lyngbya* sp.	5.02 ± 0.20^c^	664.07 ± 19.76^c^	248.39 ± 11.97^c^	21.91 ± 1.44^c^
*Microcystis* sp.	2.65 ± 0.14^b^	392.00 ± 41.71^b^	465.31 ± 25.76^d^	13.55 ± 2.76^b^
*Spirulina* sp.	1.78 ± 0.07^a^	483.33 ± 13.92^a^	213.48 ± 18.19^a^	23.86 ± 1.04^a^

a, b, c, d letters in the same column indicates significant differences (*P* < 0.05) among different species. GAEs, gallic acid equivalents; QEs, quercetin equivalents; TFC, total flavonoid content; TPC, total phenolic content

### Antioxidant activity

The FRAP assay provides a direct estimation of the antioxidants or reductants present in a sample based on its ability to reduce the ferric (Fe3^+^)/(Fe2^+^) ferrous couple (Tanvir et al. 2015). The FRAP assay is also an important indicator of the antioxidant potential of a sample. Cyanobacteria from this study produced high FRAP values for all four strains (Table 1), which is also higher than that of reported earlier (Sharathchandra and Rajashekhar 2013).

2,2‐diphenyl‐1‐picrylhydrazyl is a stable nitrogen‐centered radical that is widely used to test the free radical scavenging ability of various samples; the higher the DPPH‐scavenging activity, the higher the antioxidant activity of the sample. Scavenging activity also slightly high in *Lyngbya* sp. development of suitable antioxidant. The reducing capacity of the extract may serve as a significant indicator of its potential antioxidant activity. This activity may be due to phenolic compounds and flavonoids present in the extract as also indicated by Velioglu et al., 1998(Velioglu et al. 1998).

### Phycobiliproteins

**Figure 1:**
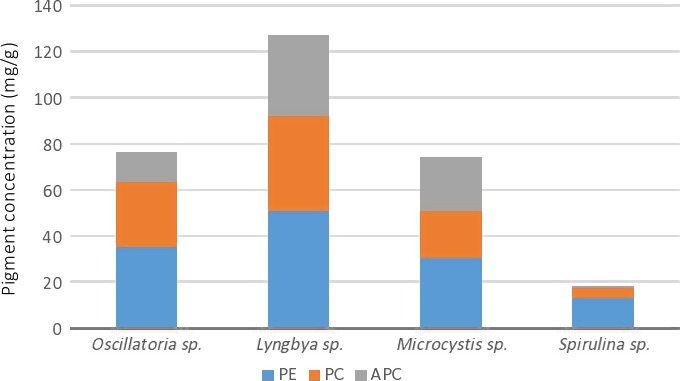
Distribution of phycobiliproteins in different cyanobacteria species.

Phycobiliproteins have been described as one of the strong antioxidants. Phycobiliproteins (PBPs) are water‐soluble pigments that can be divided into three types according to their maximum absorbance: phycocyanin (PC), phycoerythrin (PE), and allophycocyanin (APC) (Singh et al. 2005). In the study, the highest total PBPs were recorded in *Lyngbya* sp. (127.01 mg/gm) out of which phycoerythrin was dominated (51.07 mg/g) followed by phycocyanin (41.29 mg/g) and allophycocyanin (34.65 mg/g). The pigments distribution in different cyanobacteria is presented in Figure 1. The phycobiliproteins in *Lyngbya* sp. from this study is higher than *Scytonema* sp. and *Cyanosarcina* sp. but lower than *Leptolyngbya* sp. (180 mg/g) and *Phormidium* sp. (167 mg/g).

### Active compound analysis

The FTIR spectra shows that water extracts of all four Cyanobacteria species contain hydroxyl group (‐OH stretching, strong broad absorptions around the regions of ~3300–3500 cm^−1^) and carbonyl group (‐CO stretching, spectrum around ~1600–1700 cm^−1^). Only the *Lyngbya* sp.*,* does not show any absorption band around the region of ~2710–2780 cm^−1^, which are responsible for the N–H stretching. According to the antioxidant activity (TPC, TFC, DPPH), *Lyngbya* sp.*,* shows highest value than other three species. The N–H stretching band is ultimately responsible for the inhibition of antioxidant activity.

From FTIR spectra, it is evident that *Spirulina* sp. contains spectra band in the region of ~1200–1400 cm^−1^ and two weak absorption peaks which are not prominent in another three species. This weak absorption peaks are due to the alcoholic or phenolic OH groups. Therefore, Cyanobacteria *Spirulina sp*. showed lowest value with potential antioxidant activity.

## Conclusions

All four cyanobacteria species have the antioxidant activity. *Lyngbya* sp*.,* showed highest TPC, TFC, and total phycobiliproteins value. The FRAP and the DPPH radical scavenging activity was recorded highest in *Oscillatoria* sp. There were significant relationship between active compounds and antioxidant content. In FTIR spectroscopy, Lyngbya sp. does not show any N–H stretching band, which is ultimately responsible for the inhibition of antioxidant activity. The study revealed that *Lyngbya* sp. and *Oscillatoria* sp. can be excellent sources for antioxidants. Therefore, these cyanobacteria strains can be utilized as agent for providing raw materials for cosmoceuticals, pharmaceutical, and food industry.

## Conflict of interest

The author declares there is no conflict of interest exists in this study.
